# Bayesian machine learning for inverse design of ultra-high-performance concrete

**DOI:** 10.1098/rsta.2024.0041

**Published:** 2025-09-25

**Authors:** Christopher Childs, Aaron Miller, Willie Neiswanger, Barnabas Poczos, Lauren Stewart, Kimberly Kurtis, Newell Washburn

**Affiliations:** ^1^ Department of Chemistry, Carnegie Mellon University, Pittsburgh, PA, USA; ^2^ Department of Civil and Environmental Engineering, Georgia Institute of Technology, Atlanta, GA, USA; ^3^ School of Computer Science, University of Southern California, Los Angeles, CA, USA; ^4^ Department of Machine Learning, Carnegie Mellon University, Pittsburgh, PA, USA; ^5^ Civil and Environmental Engineering, Materials Science and Engineering, Georgia Institute of Technology, Atlanta, GA, USA

**Keywords:** ultra-high-performance concrete, compressive strength, machine learning, predictive model, fine aggregate, latent variable

## Abstract

The diversity of available material feedstocks, coupled with rigorous performance requirements, complicates the design of ultra-high-performance concrete (UHPC). Here, a Bayesian method for inverse design is first demonstrated from published UHPC data. Materials were represented in a framework of hierarchical machine learning; a fundamental goal in this study was to compare the accuracy and generalizability of models parameterized by compositional variables with those parameterized by latent variables based on empirical models. Data were first modelled by ensemble ridge regression, and miscalibration area (a Bayesian error metric) indicated improved generalizability for models parameterized by latent variables compared to those parameterized by composition. Then, Gaussian process regression based on an expanded feature set was used to predict strength that, counterintuitively, generated higher accuracy for models parameterized by compositional variables (test *R*^2^ = 0.91) than by latent variables (test *R*^2^ = 0.77). However, the latter more accurately predicted the properties of designs produced with untested fine aggregate and predicted novel compositions achieving high compressive strength, consistent with a significant reduction in model miscalibration error. These results demonstrate that latent variables in a Bayesian machine learning framework can provide greater generalizability across the variable space, make robust predictions on untested feedstocks and predict new UHPC compositions with optimal properties.

This article is part of the theme issue ‘Frontiers of applied inverse problems in science and engineering’.

## 1. Introduction

The compositional complexity of cementitious materials—cement types, fine and coarse aggregates, supplementary cementitious materials (SCM), admixtures and fibres—creates a high-dimensional space of input variables from which a diversity of competing performance metrics—flowability, set time, strength and durability—must be optimized under constraints of cost, feedstock availability and environmental impact [1,2]. However, the central difficulty in predictive modelling of these materials is the variability of the components; two sources of materials with the same label (e.g. CEM I, sand, fly ash) may in fact have very different effects on material properties due to the broad underlying distribution of feedstocks.

In applying machine learning (ML) and data analytics, one general solution is to use larger datasets (of order 10^3^−10^6^ samples instead of 10^1^−10^2^), which provide greater coverage over the response surface and thus should lead to more accurate models [3,4]. However, there are three issues with this approach: the first is that extremely large datasets, particularly on concrete properties collated from field testing, tend to be of lower quality than those from controlled lab settings, so that there is a greater chance of error in both the specification of compositional and processing variables and in measurement of the responses. The second related issue is that feedstocks from different sources may be labelled the same but have different chemical and physical characteristics and different effects on material properties, which complicates regression when the same nominal compositions differ in their responses. The third, less-appreciated, issue is that the variables, or features, from which models are constructed can have non-uniform predictive power across the response surface, so predictions in a limited range of compositions may not generalize, leading to overconfident uncertainty estimates. For example, predictions of material properties based solely on stoichiometry face a challenge of modelling a massive response surface for which only a small subset of data are available. In ML, this challenge can both be understood and addressed by considering ensembles of models, each of which represents a hypothesis of a prediction for a specific composition [5].

Thus, there is a critical need for a modelling framework capable of estimating the distributions of these compositional variables and accurately generalizing over a broad range of parameters, particularly when training data are sparse. Bayesian models have been applied to the design of high-performance concrete and found to be a powerful tool that provides accurate predictions of strength from a training set of over 400 samples that were characterized in terms of compositional information, such as Portland cement, blast furnace slag and coarse aggregate [6]. This work represents a state of the art for modelling, but its performance was assessed based on test/train splits of the original dataset. Thus, it works extremely well for the underlying distributions of feedstock characteristics represented in the training data, but its ability to generalize is unclear [6]. Hierarchical models that are explicitly parameterized by latent variables are a powerful tool for improving both the generalizability and interpretability of ML algorithms. Embedding physical constraints into ML models results in greater predictive capabilities, and a number of recent approaches reflect the pace of advances [7–9].

Here, the use of compositional variables is compared to latent variables in the modelling and optimization of ultra-high-performance concrete (UHPC), employing a framework of Bayesian ML [10] to explicitly account for the underlying distributions in material characteristics that conventionally make modelling and optimization so challenging. UHPC is a class of infrastructure material characterized by its high binder content, which includes SCMs and fine aggregates and very low water content, necessitating the use of high-range water reducers. These materials are defined by compressive strengths in excess of 150 MPa and post-cracking tensile strengths of at least 5 MPa [11] and often include steel fibre reinforcement to achieve those mechanical properties [12,13]. With its low water content and absence of coarse aggregate, the binder content in UHPC is roughly three times greater than normal strength concrete [14]. Today, UHPC use is increasing due to its successful translation into broad field applications ranging from precast concrete girders with similar mechanical properties to same depth-of-section steel girders [15], use in ‘closure pours’ connecting precast bridge deck panels and girders in accelerated bridge construction [16,17] and in pavement overlays [18] and structural repairs [19], among other applications. Improved UHPC design paradigms can facilitate flexibility in the selection of materials feedstocks and proportions, allowing, for example, for the use of locally available materials, emerging materials and more variable materials, each contributing potentially to increased economy and sustainability and supporting greater UHPC implementation [20,21].

Recently, conventional data analysis has been supplemented with ML methods. ML is a diverse collection of statistical algorithms that are utilized to predict a system’s properties, which is starting to be used widely in infrastructure materials, including UHPC [22,23]. For example, Ghafari *et al.* [24] trained an artificial neural network (ANN) on 53 different UHPC compositions, optimizing a composition that experimentally agreed with predicted compressive strengths within 5%. Although the optimized blend did not extrapolate to predicting an optimized blend outside of the range of tested compositions, the ANN outperformed traditional statistical mixture design approaches based on multiple linear regression. However, this ANN was parameterized by the specific materials in the training set, which limited its predictions to these components. For each various material with a different size, surface area or reactivity, there would be a lack of generalizability for this model, and additional experimentation would have to be performed to retrain an ML algorithm.

One method for improving generalizability, particularly for small datasets, involves representing the system in terms of latent variables, or variables that are not directly observed or measured but represent underlying factors governing performance [25,26]. Based on the existing literature for optimizing blends based on particle packing and water film thickness (WFT) equations, a hierarchical ML (HML) model [27–29] is presented here for the prediction and optimization of UHPC compressive strength. Data from literature were encoded with latent variables based on particle packing (as quantified by the compressible packing model (CPM)) [30] and initial free water (defined by WFT). This approach allows for generalization to untested materials of various sizes, reactivities and surface areas. A validation set of UHPC samples designed and created from a disparate source of materials predicts UHPC compositions of high compressive strength.

## 2. Latent variables governing ultra-high-performance concrete properties

UHPC strength develops through chemical, physical and mechanical mechanisms, and while the principles are generally understood, the complexity of the compositional parameter space makes designing for strength challenging. The improvement in chemical strength is due to the pozzolanic activity induced through the utilization of SCMs. SCMs are amorphous materials composed of silica and alumina, which do not react as cementitious materials. Instead, these materials, which include silica fume, metakaolin and fly ash, react with latent CH from hydrated Portland cement. This pozzolanic reaction is responsible for the formation of strong C–S–H and C–A–H amorphous phases [31]. In UHPC, both metakaolin and silica fume are widely studied materials due to their high purity and high specific surface areas (SSAs), which promote the pozzolanic reaction [32,33].

The underlying physical improvement in UHPC strength is due to the minimization of porosity in the microstructure [34]. In UHPC, there are several types of void formation. The first is interlayer spacing within the C–S–H and C–A–H phases. The size of these pores is between 5 and 25 Å and, as such, is within the realm of van der Waals interactions; therefore, they are not detrimental to cement strength [35]. A second type of void formation is capillary pores. These pores have been found to be inversely related to cement strength, and the size and continuity vary directly with the water-to-cement (w/c) ratio [36]. Finally, void formation is found in the interfacial transition zone (ITZ) between the hydrated cement paste and aggregate phase in UHPC. Generally, the ITZ is considered as the strength-limiting phase in concrete [35]. Because the ITZ width scales with aggregate size, only fine aggregates (i.e. sand and flours) are used in UHPC [37]. Furthermore, the void formation within the ITZ can be reduced through the utilization of pozzolanic, high surface area SCMs, which lead to increased chemical bonding and physical interactions through increased packing, respectively [38,39]. Although no fully comprehensive model exists between the complex interactions and correlations between these mechanisms, this paper serves to disentangle these interactions through ML modelling on these mechanistic features.

A traditional methodology to minimize the void ratio in the ITZ for UHPC is through maximization of particle packing. Two common models for optimizing UHPC compositions include the modified Andersen and Andreasen model and the CPM. The modified Andersen and Andreasen model [40] incorporates particle size distributions and an adjustable parameter, *q*, to generate an ideal gradation curve where actual compositions can be manually fit with the *q* parameter to find an optimal packing density. The CPM was first developed by de Larrard and calculates a packing index, *K*, which can be optimized to a specified value, where *K* = 4 is a suggested value for a self-consolidating concrete mix [41].

A second factor in minimizing UHPC porosity is having a low w/c ratio. This ratio decreases the formation of capillary pores by limiting the amount of unreacted water in the system [34]. While packing density is solely based on the solid content and particle diameters of the mixture, the WFT parameter considers the water content and surface areas available for water adsorption [42]. Even with an optimized packing density, excess water could lead to capillary pore formation. While increasing particle packing leads to an increase in compressive strength, an increase in WFT leads to a decrease [43]. Depending on the size and surface area of solids, along with the w/c ratio, a complex interplay occurs between attempts to optimize particle packing, WFT and SCM reactivity.

Discontinuous fibre reinforcement is an additional strategy to improve the mechanical performance of UHPC. Steel fibres are commonly added at 2–3% but can be as high as 6% [44], and they serve to reduce the brittle behaviour of the material. Under an external load, these fibres can control the formation of microcracks or influence crack propagation, and their toughening effects lead to an increase in the compressive, flexural and tensile strength [45].

## 3. Experimental

Bayesian ML models were trained on data curated from the literature. These datasets were selected based on the following criteria:

(1) All compositions are designed to achieve strength consistent with UHPC.
(2) Contained no coarse aggregate, as defined as the mean D50 aggregate with a threshold of 600 μm (i.e. #30 sieve size) being the largest particle size modelled.
(3) All fine aggregate materials had a measured D50 to properly represent the middle layer in the hierarchical model.
(4) Contained samples cured around room temperature to preclude mechanisms of strength gain in steam and heat curing methodologies.

Following the training of the models based on literature data, separate experiments were performed in the laboratory to validate some of the optimization predictions of the GPR model using the materials and methods described here.

### (a) Materials

For the validation experiments, cement mortars were prepared using an ASTM C150 Type I Portland cement (Lafarge Holcim, Duluth, GA), metakaolin (MetaMax, BASF) and silica fume (Elkem Materials, Inc.), as well as subangular sand (River Sand Inc., Buford, GA) with a mean particle diameter of 600 μm. Also included as part of the compositions were a polycarboxylate ether high-range water reducer (MasterGlenium 7920, BASF) as well as steel fibres (Dramix, Bekaert) with a 13 mm length and 0.20 mm diameter. The composition of the cement was 58% C_3_S, 18% C_2_S, 2% C_3_A, 13% C_4_AF and 2.5% CCbar with a Blaine fineness of 394 m^2^ kg^−1^.

### (b) Assessment of strength

All mixes were prepared in 850 cm^3^ batches in a countercurrent (Hobart C100), 9.5 l-capacity mixer with a paddle attachment. The following mixing procedure was adapted from several papers on UHPC mix development [12,46,47]. First, oven-dry sand and SCMs were mixed on low speed for 2 min. Then, cement was added and mixed on low for 1 min. Water was gradually added over 30 s while mixing on low for an additional 30 s. Next, superplasticizer was added, and the mixture was mixed on low for 10 min. Afterwards, the material’s flow was tested, and, if satisfactory, specimens were cast. If further adjustments to flow were required, superplasticizer was added in 2 ml doses and mixed for an additional 2 min per dose until the mix achieved a flow of 9 inches or more. The mix was then repeated following the same procedure but with the adjusted amount of superplasticizer. This method ensures that all mixes were evaluated with the same mixing time. Superplasticizer was added after the water because it has been observed that delaying its addition increases the fluidity of self-consolidating concrete and UHPC [48,49]. Additional time was allowed for the mix to cohere before fibres were added, just before casting. For each mix, six replicate mortar cubes (5.08 × 5.08 cm) were cast for compression testing. After curing in a 23°C limewater bath, tests were performed at 7 and 28 days, with load applied to cast (unfinished) surfaces at a rate of 136 kg per sec [50].

### (c) Data collection

For model training and testing, a database was compiled of UHPC mixtures from published literature [24,33,34,51–68]. These are compiled in a published dataset [69]. Four datasets were chosen for training the model and are summarized in table 1. To validate the samples on in-house prepared UHPC compositions, the collected datasets were limited to those cured at 20**°**C with 28-day compressive strengths in excess of 100 MPa. A strength lower than the lower bound for conventional UHPC was chosen to expand the performance space for the training dataset, allowing a broader range of predictions as well as accommodating greater variability in design parameter values. Table 2 lists the ranges of each of the UHPC constituents combined amongst all four papers. From tables 1 and 2, it can be seen that these data are largely representative of common compositions for UHPC mixtures. However, while silica fume, metakaolin (or relatively pure calcined clay) and fly ash are among the most commonly used SCMs in concrete, as well as UHPC, other SCMs (e.g. slag, relatively impure calcined clay) are not included in this dataset. This could limit generalizability, particularly if SCM reactivity deviates considerably from that included in the training set. However, slag is considered in the equivalent cement content used in the latent variable training set, as described in §4a(i).

**Table 1.** Datasets compiled for training.

data source	Tafraoui *et al.* [33]	Ghafari *et al.* [24]	Berry *et al.* [34]	Wille *et al.* [61]
**#** samples	7	50	41	7
SCMs	silica fume metakaolin	silica fume	fly ash silica fume	metakaolin
fine aggregates D_50)_	sand—230 μm quartz—11 μm	sand—400 μm quartz—7 μm	sand—500 μm	sand—110 μm sand—500 μm glass—5 μm
fibres	steel fibres, 13 mm in length and 0.16 mm in diameter	two types of steel microfibres with diameters/lengths of 0.2/0.15 and 13/10 mm	no fibres utilized	smooth, hooked and twisted fibres ranging from 0.12 to 0.3 mm in diameter and 6 to 30 mm in length

**Table 2.** The ratios of each constituent material for UHPC.

number	variable	range
1	mass of cement-to-mass of solids	0.29−0.50
2	mass of metakaolin-to-mass of solids	0−0.11
3	mass of fly ash-to-mass of solids	0−0.13
4	mass of silica fume-to-mass of solids	0−0.12
5	mass of sand-to-mass of solids	0.30−0.54
6	mass of quartz-to-mass of solids	0−0.23
7	water-to-cement ratio	0.17−0.29
8	volume fraction of steel fibres	0–3.5%

Because mixing method, including mixing energy, order of addition and timing, along with mixer type and volume, are all particularly important for achieving homogenization of UHPC, variations in mixing methodology among the data sources should be noted. Among the four sources used for training data, detailed mixing procedures are provided for three [20,30,44], and some information is provided for the other [31]. For those providing details, all mixed dry ingredients first and then added water and superplasticizer, followed later by fibre addition; the procedure used in the preparation of test mixtures reported here follows this process, as detailed in §3b. However, the type of mixer used varied among these sources, with [30] using a 10 l-capacity countercurrent mixer (similar to the mixer used here), while [31] and [44] used both a horizontal mortar mixer and an industrial ‘cake mixer’ (of unstated capacity, but likely similar in mixing action to the countercurrent mixer). In [20], the mixer type is not specified. While extensive mixing times have been reported in some early UHPC studies, with the procedures outlined above, homogenization can typically be achieved in under 20 min [30].

## 4. Computational methods

### (a) Data representation

The amount of cement, sSCMs, filler materials (e.g. quartz), aggregate (i.e. sand), water, superplasticizer and steel fibres in each unit volume of mix was recorded. Additionally, the curing temperature and 28-day compressive strength results of each mix were included as the output for the dataset. All reported mix design parameters from the training samples were converted on a per mass basis of the whole mixture (solid and water phases) utilizing an assumed specific gravity for each phase. The reported average particle diameter (D50) and SSA from each data source for the fine aggregates were utilized. However, for SCMs where particle sizes were recorded, the values based on conventional characteristics for reach were assumed, as listed in table 3. These are based on the measurements of materials utilized for the testing at Georgia Tech.

**Table 3.** Measured particle parameters for UHPC components.

particle	specific gravity	D50 (μm)	SSA (m^2^ kg^−1^)
cement	3.15	15	394
fly ash (class C or F)	2.38	25	500
silica fume	2.22	0.2	18 000
metakaolin	2.3	12	14 000
sand	2.5	varies by source	varies by source
quartz	2.65	varies by source	varies by source

**Figure 1.**
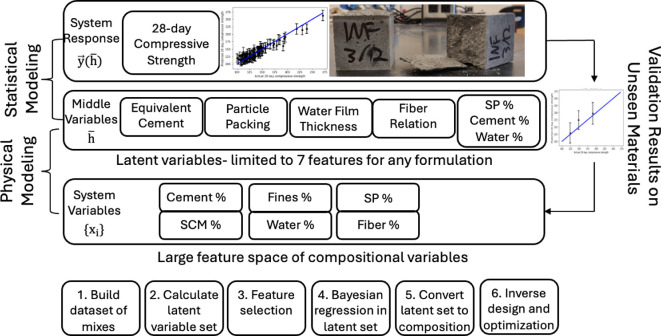
Schematic of the HML model for UHPC compressive strength. The bottom layer represents the compositional space for UHPC, which would be a common parameterization for an ML model to predict material properties. In HML, a middle layer represents latent variables that govern the underlying forces and interactions that determine the system property being modelled. This middle layer can be used to build a second response surface and is related to the output, the 28-day compressive strength, via feature selection and regression, which can then be optimized to predict high-strength UHPC blends for any arbitrary set of compositional materials. The steps in building an HML model are listed below the schematic.

Four parameters were chosen for inclusion as latent variable representations of the composition in the model: the equivalent cement content, the particle packing of the mixture, the WFT and an empirical relation of fibre addition to compressive strength. In addition to these latent variables, three compositional features that are common in any UHPC blend were included: the superplasticizer content, the water content and the cement content. These parameters were selected for consideration based on knowledge of established relationships in cement to direct the HML model from compositional to middle-layer variables, as shown in figure 1.

#### (i) Equivalent cement content

The concept of an ‘equivalent cement’ value first appeared in work on the thermal control of mass concrete placements [70]. In the current application, it serves as an estimate of the approximate amount of heat generated by hydraulic reaction, producing strength-giving products (e.g. C–S–H, C–A–S–H), and thus is expected to correlate with compressive strength. In equation (4.1), each mix component was normalized into an ‘equivalent’ weight of cement based on its contribution to the heat of hydration during the first 28 days. For example, since Class F fly ash is pozzolanic and slower reacting compared with regular Portland cement, the amount of Class F fly ash is multiplied by 0.5 as shown in equation (4.1). This concept was incorporated as domain knowledge as a way of gauging the reactivity of a mix in the absence of calorimetry data, with the relative contributions of each SCM to hydration heat.

(4.1) Equivalent Cement=Cement+0.5×(Class F Fly Ash)+0.8×(Class C Fly Ash)+1.2×(Silica Fume)+1.2×(Metakaolin)+X×(Slag)

Note that the value for the slag contribution depends on the amount of replacement for cement, with *X* = 1.1 (0–20% replacement), 1.0 (20–45% replacement), 0.9 (45–65% replacement) or 0.8 (65–80% replacement).

#### (ii) Particle packing

Including particle packing as a latent variable requires an input parameter characterizing the initial water-filled porosity in a single parameter. The packing model is based upon the CPM, which has been demonstrated to be well-suited for multi-component, polydisperse systems, and is considered foundational to the design of UHPC [71–73]. The CPM summarizes the packing of the mixture into a single parameter, *K*. Higher *K* values correspond with denser mixtures and higher compressive strengths. Particle packing is also considered to be a critical factor in determining the material properties of cement in both the plastic and hardened states, and it can be used as a design variable in increasing the loading of fine aggregate and SCMs and controlling the properties of these materials. The components of calculating K are given by:


(4.2) $$a_{ji}=\sqrt{1-{\left(1-\frac{d_{j}}{d_{i}}\right)}^{1.02}}$$

(4.3) $$b_{ji}=1-{\left(1-\frac{d_{i}}{d_{j}}\right)}^{1.5}$$

(4.4) $$\Phi_{i}^{*}=\beta_{i}\left[1-\sum_{j=1}^{i-1}\left(1-b_{ji}\left[1-\frac{1}{\beta_{j}}\right]\right)\Phi_{j}-\;\sum_{j=i+1}^{n}\frac{a_{ij}}{\beta_{j}}\Phi_{j}\right]$$

(4.5) $$K=\;\sum_{i=1}^{n}\left(\frac{\frac{\Phi_{i}}{\Phi_{i}^{*}}}{1-\;\frac{\Phi_{i}}{\Phi_{i}^{*}}}\right),$$

where

*d*_*i*_ = grain size of rank *i*
*d*_*j*_ = grain size of rank *j*
*a*_ij_ = coefficient for the loosening effect, exerted by the grains of rank *j* on those of rank *i*
(*j* > *i*)
*b*_ji_ = coefficient for wall effect, of the grains of class *i* on the grains of rank *j* (*j* < i), with
*d*_1_ > *d*_i_ > *d*_n_
*Φ^∗^* = maximum possible volume in the presence of other particles
*Φ* = volume of particles present
^*β*^ = virtual packing density
*K* = packing index, a unitless number that relates to packing.

Strength was hypothesized to vary with packing index (*K*), making it a potential latent-variable feature for the model. Based on particle size distributions for each of *n* components, the loosening (*a*_ij_) and wall (*b*_ij_) coefficients are determined and used to calculate the maximum possible volume for each particle size (*Φ*_ι_*), similar to study [74], suggesting particle packing models can be used to predict flow and strength, particularly at early ages, in these systems. A Python script was created to represent each blend without explicitly measuring the actual packing density, and *ф* was calculated as (1-water fraction), while *β* was held constant for each UHPC blend.

#### (iii) Water film thickness

The WFT is a relationship between the amount of water present in the mixture and the particle surface area. In conventional concretes, a higher WFT value corresponds with higher workability [42]. To achieve the necessary strengths for UHPC, it is necessary to use minimal amounts of water in the mix, even less than what may be necessary to accomplish complete cement hydration, to minimize the initial intrinsic porosity.

In a cementitious system, the pore solution phase is divided into two distinct types [75]. The first, filling water, is the water that fills voids within solid particles and does not contribute to workability. The second type, after these voids have been filled, is the excess water, represented in equation (4.6) [76]


(4.6) $$u_{w}^{\prime }=u_{w}-u,$$

where

$u_{w}^{`}$ = excess water
$u_{w}$ = ratio of water in system by volume
u = voids ratio (the ratio of the volume of voids to volume of solids in the composition).

The amount of excess water is divided by the SSA weighted on the volume fraction of all the particles in order to determine the WFT, as shown in equation (4.7)


(4.7) $$WFT=\frac{u_{w}^{\prime }}{A_{m}},$$

where

WFT = water film thickness
$A_{m}$ = specific surface area.

WFT has been shown to correlate well with cement paste rheology and strength [43], making it a potentially important latent variable for UHPC. However, unlike the CPM, the average SSA of cementitious particles is also considered, and an increase in surface area leads to a corresponding decrease in WFT [42,75]. As the actual packing density, *ф*, for these blends was not measured, the voids ratio, u, was calculated as shown in equation (4.8)


(4.8) $$u=\frac{\left(1-ф\right)}{ф},$$

where

ф = (1−water content).

This allows for a consistent way to represent *ф* without directly measuring the extent of packing density for each blend.

#### (iv) Superplasticizer content

While superplasticizer imparts workability to UHPC, an excess of superplasticizing admixture can cause the strength development to be delayed [77]. Thus, it is important for the HML model to consider the amount of superplasticizer present and balance workability with strength development. Superplasticizer content was not related to a latent variable representation in WFT of particle packing, and for these reasons, this bottom-layer compositional parameter was represented as a middle-layer parameter to help the model understand a tradeoff in requiring sufficient superplasticizer to have a workable blend.

#### (v) Fibre relation

Generally, UHPC contains 2–3% steel fibres by volume [78]. The fibre content was not related to a latent variable representation in WFT or particle packing, and for these reasons, an empirical relation presented in Siwinksi *et al.* [79] was used to relate the amount of fibre in a UHPC composition to the relative increase in compressive strength and is determined as


$$k_{fr}=e^{0.034*p_{s}},$$

where *k_fr_* is the reinforcing-fibre coefficient and *p_s_* is the percentage ratio of steel-fibre mass to the mass of cement. The fibre relation *k_fr_* is a latent-variable representation of fibre loading used in the middle layer. While fibres of different sizes and shapes were used in the training data, these were all represented simply by the mass fraction.

### (b) Machine learning models

Bayes’ theorem determines the posterior probability of an event based on the probabilities of the factors constituting the event—prior probabilities and likelihood of these occurring. Most approaches to ML provide a maximum likelihood estimate in the predictions of system properties or responses, but it can be difficult to gauge the quality of the predictions except by direct validation. In explicitly estimating the underlying statistical distributions, Bayesian predictions have associated mean and variance values, which can provide a powerful tool for understanding and optimizing complex systems. In this work, two Bayesian approaches were used to model data on UHPC strength. The first approach provided an alternate metric known as miscalibration area [80] to assess the quality of compositional models versus those based on latent variables, in addition to the more traditional root mean squared error (RMSE) or *R*^2^ metrics. The second was used for modelling, physical interpretation and optimization. Together, these two approaches demonstrate the power of Bayesian methods in modelling cement and concrete.

In the first approach, we estimated this posterior distribution for Bayesian optimization (BO) to compare the uncertainty of models parameterized by compositional variables (bottom layer of HML) with those parameterized by latent variables (middle layer of HML). At each iteration in BO, an approximation to the posterior probability density function can be produced by sampling from this posterior distribution. The approach of utilizing Bayesian analysis, which involves marginalizing over the posterior distribution of parameters, is to yield a better prediction result both in terms of accuracy (models that predict similar values to training data) and generalization capability (predictions of the compressive strength of new compositions in validation sets). Error analysis takes place through comparing the RMSE, a prediction score that ignores Bayesian probability and compares how well the mean values of the data fit to the best model, and the miscalibration area, a quantification of uncertainty in the model based on calibration techniques developed by Kuleshov *et al.* [81]. Standard Bayesian uncertainty estimates, such as those generated by Gaussian process regression (GPR), often underestimate the true uncertainty—for example, when the 90% credible interval does not contain the true outcome 90% of the time—due to model bias, as when the model features do not have constant predictive power across the domain of the outcome being modelled. The miscalibration area was developed to provide a more accurate framework to quantify uncertainty and provides an alternate assessment of model quality.

Miscalibration error utilizes a predictive uncertainty method that makes a prediction and gives an uncertainty in the form of an ‘X% credible interval’, which aims to capture the true point X% of the time. A hold-out test set is then utilized to measure how many test points the credible intervals contain the true point. By performing this hold-out test for every X% between 0 and 100%, an average difference between the goal percentage and the measured percentage (averaged over each goal percentage value from 0 to 100%) can be computed, giving the miscalibration area. This Bayesian metric relates to how well the uncertainty errors capture where the values should be. Hence, the model learns if it is predicting well or poorly to each test point based on a given set of features. Here, this approach will be used to explore the tradeoffs in parameterizing complex systems by latent variables instead of compositional variables.

In the first type of approximate Bayesian inference, we used a probabilistic ensemble model comprising 20 ridge regression models (i.e. linear models with L2 regularization) parameterized separately by the bottom layer and middle layer of the training data. Each element of the ensemble was instantiated with a randomly drawn regularization strength and initial random state. After training each ensemble element on a given dataset, the given values of compositional or latent variables were taken as parameters of a Gaussian approximation to a posterior distribution over functions of that input. This resulted in the model for predicting both the mean and the variance of each point in the dataset. To extend the predictions to validation, a monotonic transformation of the posterior variance parameter was applied, calculated from the values of the dataset from Wille *et al*. [61], which produces a modified posterior approximation with improved average calibration.

In the second type of Bayesian modelling, the feature space of the middle layer was first expanded by including cross-terms, defined as the products of the latent variables, and feature selection was used to reduce this set to those that were most strongly correlated with the compressive strength and thus hypothesized to represent the latent variables that govern the properties of the material. (Cross-terms can be used to represent interactions between variables in the model.) The data were randomly split into 80% train and 20% test sets. Variable selection in decomposing the compressive strength into contributions from the physicochemical forces and their cross-products was performed using Lasso [82] (i.e. regularized regression with an L1 norm) and tenfold cross-validation, but feature selection was not used in the compositional representation of the response surface. In addition to improving the accuracy, feature selection from latent variables improves the interpretability of the model based on the middle layer [27,29].

A GPR was then utilized as the ML technique in the prediction of UHPC compressive strength from the features selected by Lasso. This model was also compared directly with that trained on the bottom-layer compositional space for comparison. GPR is a Bayesian methodology that can be utilized to learn both the predicted mean and posterior probability for the expected range of error at each prediction. GPR utilizes a metric of distance known as a covariance function (or kernel) to learn the distribution of functions over the training data [83]. From a prior establishment of the mean and covariance function, GPR finds a posterior distribution based on the training data. Instead of utilizing a cross-validation approach as is common in many ML methodologies, GPR updates hyperparameters in the covariance function through an optimization procedure on the log marginal likelihood [84]. The strength model was trained with the output, and each input was standardized utilizing the StandardScaler methodology from scikit-learn, and GPR was performed utilizing GaussianProcessRegressor in scikit-learn modelling with a standard radial basis function kernel [85]. The data were trained and evaluated on 80% train and 20% test sets as employed in the Lasso model.

## 5. Results and discussion

### (a) Ensemble ridge regression and Bayesian optimization

**Figure 2.**
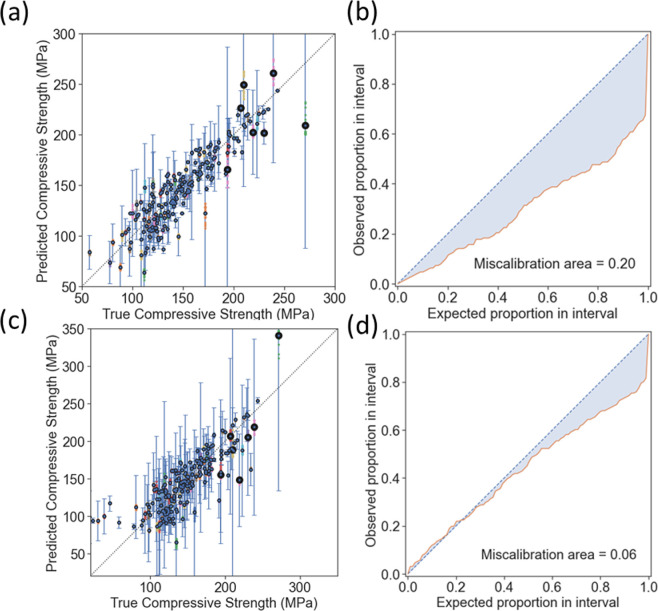
Results of the regression showing (a) the predicted and actual values, and (b) miscalibration area utilizing the bottom-layer compositional variables as inputs. Results of the regression showing (c) the predicted and actual values, and (d) the miscalibration area utilizing the six middle-layer variables as inputs. The points with the larger dark circles represent the validation dataset (Wille *et al.* [61]), having RMSE values of 34.0 MPa for compositional parameterization and 43.0 MPa for parameterization by latent variables.

The results for the uncertainty ensemble utilizing a ridge regression for the bottom layer (figure 2a,b) and middle layer (figure 2c,d) are shown below. The MSE, RMSE and miscalibration areas are tabulated in table 4.

**Table 4.** Statistics representing the bottom and middle layers MSE, RMSE and miscalibration area for the test dataset.

	MSE	RMSE (MPa)	miscalibration area
bottom layer	424	20.6	0.20
middle layer	660	25.7	0.06

In comparing the parity plots shown in figure 2a,c, it is apparent that the mean values for each sample cluster around the 45° lines in both, suggesting that there is no systematic bias in the models towards overestimating or underestimating compressive strength. The RMSE, represented as the error bar on each point, varies across data points but is often a significant fraction of the expected value, which is attributed to the size of the dataset and the relatively sparse compositional and latent-variable models that were used to build models. The RMSE of the uncertainty model parameterized by the compositional variables was smaller than that parameterized by the latent variables (20.6 versus 25.7 MPa, respectively), consistent with a model with lower uncertainty as calculated based on the posterior distribution estimate from the ensemble ridge regression.

The data from Wille *et al.* [61] were used as a small, internal validation set containing seven compositions, including sand with a smaller value of D50 (110 μm) than any of the training data, as well as glass beads with D50 of 5 μm. The predictions of the compositional model had an RMSE of 34.0 MPa, while that for the latent-variable model was 43.0 MPa, and it is observed that points in the validation set tend to cluster more uniformly around the 45° line in the parity plot in the former, while they appear to be below this line in the latter.

Based on this analysis, it appeared that the compositional model had a greater accuracy than the latent-variable model. However, in exploring the miscalibration area plots shown in figure 2b,d, it is seen that the compositional model had a significantly worse calibration than the latent-variable model (0.20 versus 0.06, respectively). That this area fell almost entirely below the 45 line indicates that the expectation value for the uncertainty was greater than the observed uncertainty, from which it is concluded that the compositional model significantly *underestimated* the actual uncertainty of the response surface parameterized by the bottom layer of the HML model. Indeed, while the traditional RMSE metric suggests that compositional parameterization is suitable for optimization, the miscalibration area suggests that it may not accurately predict novel compositions with high compressive strength due to low generalization power at compositions outside the range of the training set. While BO can be used for this purpose, we instead explored GPR following Lasso feature selection from an expanded set of latent variables as a means to provide optimized compositions with greater interpretability for the forces and interactions that govern the compressive strength of UHPC.

### (b) Feature selection through Lasso

**Figure 3.**
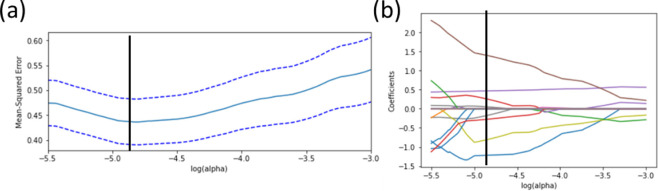
(a) Cross-validation plot for Lasso showing the best selected regularization parameter, as indicated by the black line at alpha = 0.008. (b) Coefficient paths at each regularization parameter. At the optimal alpha value, there are eight remaining features determined as important to the model.

The middle layer from the model initially consisted of seven features: equivalent cement, packing density, WFT, empirical equation for fibre and the water reducer, cement and water percentages in the UHPC composition. These features were appended with cross-terms from these primary variables for a total of 28 features. Upon cross-validation (figure 3a), an optimal regularization parameter of alpha = 0.008 was selected. This resulted in a final feature space dimensionality of 8, as shown in the coefficient plot in figure 3b.

**Figure 4.**
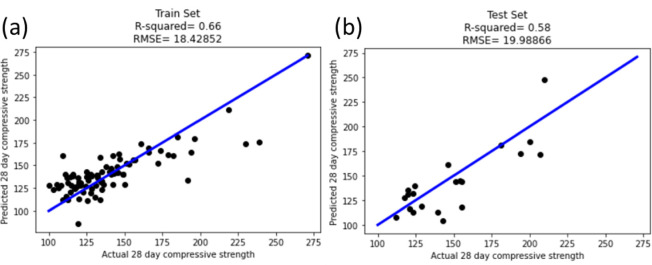
Parity plots for Lasso regression results performed for (a) training (*R*^2^ = 0.66) and (b) test sets (*R*^2^ = 0.58).

In figure 4, the results for the Lasso model are presented, showing the RMSE and *R*^2^ values for both the training and test sets. While the accuracy of models based on linear regression may not accurately capture the shape of the response surfaces, they do offer facile interpretability, particularly for models parameterized by latent variables.

Based on balancing accurate predictions of the data in the training and test sets in the Lasso analysis, the final feature set was determined to be composed of the equivalent cement, fibre equation and the cross-terms between equivalent cement*packing density, equivalent cement*WFT, packing density*WFT, packing density*water%, water reducer%*cement% and fibre equation*cement%.


28dayCompressiveStrength=

−1.22∗Equivalent cement

+ 0.47∗Fibre relation

− 0.16∗Equivalent Cement∗Packing Density

−0.80∗Equivalent Cement∗WFT

+0.24∗Packing Density∗WFT

+0.05∗Packing Density∗Water%

−0.27∗Water Reducer%∗Cement%

+1.39∗Fibre Equation∗Cement%

This equation represents the response surface describing the UHPC compressive strength as a polynomial function of these latent variables and select cross-terms. The model assumes that the hypersurface defined by the data in the training set is a linear function of these variables, and thus both the training and test *R*^2^ values are relatively low (0.66 and 0.58, respectively). Here, a positive coefficient represents a variable that is positively correlated with the value of compressive strength, and the magnitude of the coefficient represents the relative contribution of the variable. Linear terms represent the impact of a single latent variable on compressive strength, while cross-terms, which comprise the bulk of the model features, reflect coupling between variables that may have complicated interpretations. In reviewing the coefficients in this sparse model, most terms are consistent with the understanding of the factors that contribute to UHPC strength. For example, the positive coefficient of ‘fibre equation’ represents increases in strength with fibre loading, and the positive coefficient on two cross-terms involving packing density is also consistent with the design principles.

The terms involving ‘equivalent cement’ are less clear. These represent pozzolanic activity, and the linear term and the two cross-terms involving the product with packing density and WFT all have negative coefficients. One interpretation of these terms is that the algorithm is modelling a limited parameter space of composition and thus latent variables. While it is well established that higher pozzolanic activity is necessary for higher strength, there may be tradeoffs with other factors that lead to the negative correlations represented here, such as correlations between compressive strength and higher levels of replacement of cement by silica fume, metakaolin and fibres. Thus, these trends may only be valid in the relatively narrow space of equivalent cement investigated here. This serves as a reminder that ML in these applications is primarily a tool for design and optimization via interpolation within a range of parameters.

Furthermore, regularized regression is a useful method for feature selection, but the variables identified as strong determinants of strength can be used in more powerful algorithms that fit the response surface more accurately by capturing the interplay between variables. GPR is one tool that finds broad applicability in science and engineering, and it was applied here.

### (c) Gaussian process regression

In GPR, the data in the training set are used to build a more robust model of the response surface than can usually be accomplished using regression. While the approach of regression is to develop a function that predicts the value of individual data points on a response surface with minimum error, GPR uses correlations between data points in developing this model. An additional hyperparameter in these models is the length scale over which correlations are considered, which allows the algorithm to adjust predictions smoothly with a large length scale or to allow rapid changes between neighbouring points with a small length scale. This flexibility allows GPR to model a diversity of physical systems.

One additional advantage of GPR is that the algorithm automatically provides an uncertainty estimate for each predicted point. Some points on the response surface may be well fit by the model with a low variance, while others may have higher estimated uncertainties. Because different combinations of variables—either compositional or latent—can result in similar predictions, points with similar strength values can have very different predicted uncertainties. Although the mathematics behind GPR is complicated, it provides a powerful tool for modelling complex physical systems [86]. Here, GPR models were developed based on compositional variables (bottom layer) and latent variables (middle layer) to compare the predictions and their utility in optimization.

**Figure 5.**
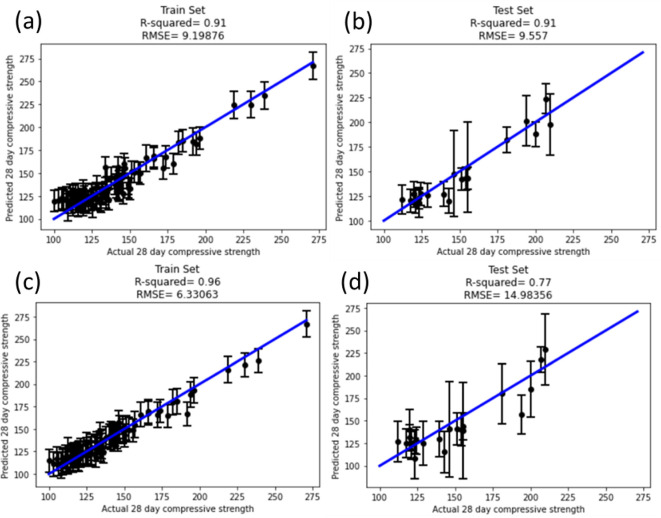
GPR results performed using the compositional (bottom) layer features for the (a) training and (b) test sets. GPR results performed using the middle-layer features for the (c) training and (d) test sets.

The results for the GPR model for the bottom layer (figure 5a,b) and the final eight-feature middle layer, as determined by Lasso (figure 5c,d), are shown below. The *R*^2^, MSE and RMSE are shown in table 5. It is interesting to note that the train and test accuracy values are similar for the compositional models (both 0.91), but the test accuracy for the latent model is somewhat lower than the train value (0.77 versus 0.91, respectively). This suggests that the predictions of similar compositions are more accurate than the predictions based on latent variables, which is also observed in the larger error bars in more of the predictions in figure 5b.

**Table 5.** Statistics representing the bottom and middle layers, *R*^2^ and RMSE.

	*R* ^2^	RMSE (MPa)
bottom layer—train	0.91	9.2
bottom layer—test	0.91	9.6
middle layer—train	0.96	6.3
middle layer—test	0.77	15.0

The bottom-layer and middle-layer parameterization resulted in RMSEs of 9.6 and 15.0 MPa on the test sets, respectively. While the bottom-layer regression outperformed the middle layer by slightly over 5 MPa in terms of RMSE, utilizing the middle-layer parameterization allows for generalizing to a UHPC composition of untested material characteristics. Training on the high-dimensional compositional feature space provides for RMSE <10 MPa when interpolated within the model. However, this can be extended to new feedstock materials utilizing the latent representation, where each composition can be represented across a more uniform distribution [87]. This represents a simple form of transfer learning to compositions that were not explicitly contained within the original dataset.

### (d) Generalizability performance on a validation set

To demonstrate generalizability, a new validation set of three unique UHPC compositions was produced and tested. A different size sand (600 versus 500 μm) than that within the training set was utilized, along with a different source material, limestone. The proportions and measured compressive strengths of these compositions are listed in table 6.

**Table 6.** Proportional mix designs (by weight of cement) for the validation set along with the measured 7-day and 28-day compressive strengths.

	mix A-1	mix A-2	mix A-3
cement	1	1	1
silica fume	0.219	0.255	0
metakaolin	0	0	0.206
steel fibres	0.284	0	0.245
water	0.226	0.201	0.215
superplasticizer	0.025	0.023	0.021
river sand—600 μm	1.581	1.500	1.378
limestone—45 μm	0.215	0.071	0
curing temperature	20	20	20
measured 7-day strength (MPa)	134.6	96.8	125.8
measured 28-day strength (MPa)	189.9	123.6	148.7

**Figure 6.**
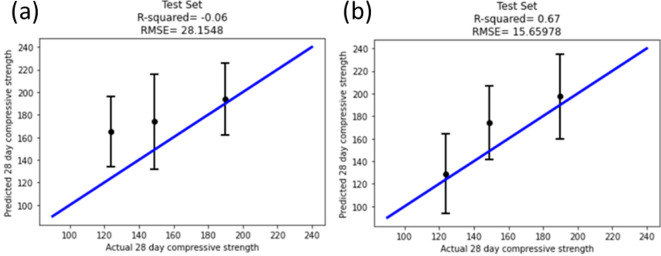
(a) The GPR model from the bottom layer. River sand and limestone were placed in the features that most closely matched the sizes of these validation compositions. (b) The GPR model utilizing the middle-layer representation. This feature space does not rely on representing various-sized materials as a different feature but is generalizable to an arbitrary compositional space that follows similar latent variables.

These validation compositions were applied through both the bottom- and middle-layer-trained GPRs to establish the generalizability of each model. Two new materials were introduced into the validation compositions: river sand with a D50 of 600 μm and limestone with a D50 of 45 μm. To utilize the GPR trained on the bottom layer consisting of features from the compositional space, the materials closest in size were utilized as the feature representing the material. The compositional percentage of river sand was placed in the feature from the training set representing sand with a D50 of 500 μm. Limestone was placed in the feature from the training set representing crushed quartz with a size of 11 μm. For the middle-layer representation, the feature space is composed of a latent representation of compositional space without specific sizes encoded. Based on the compositional space of the validation set, the particle packing, WFT, equivalent cement and fibre relation were calculated. The results from training with each model are shown in figure 6.

Despite the bottom-layer GPR model showing improved performance on the test set, there was a decrease in the *R*^2^ from 0.91 to −0.06 and an increase in RMSE from 9.6 to 28.2 MPa on the validation set. The middle-layer representation shows a decrease in *R*^2^ from 0.77 to 0.67 and an increase in the RMSE from 15.0 to 15.6 MPa, demonstrating consistency with what would be expected when generalizing beyond the training and testing datasets. For models parameterized by the middle layer, there is a higher expectation that a higher proportion of samples will have a value that will fall within the predicted uncertainty of the model.

### (e) Future directions

The disparate types of data utilized for predicting the properties of cementitious materials have inherent uncertainties that reflect the underlying uncertainties of compositional variables. The Bayesian framework presented here could form the basis for advanced analytics capable of integrating a diversity of data on cement, SCMs, aggregate, fibres and other constituents to optimize the properties of complex mixes such as UHPC. While the size distribution of fine aggregate is an important characteristic in mortar and concrete, this is one of many features that can be incorporated into this framework. As more powerful measurement tools provide detailed information on shape, composition, pore structure and crystallinity, Bayesian ML can be used to leverage the theoretical and empirical relationships developed in modern research in cement and concrete to predict complex behaviours, such as rheology, strength development and durability.

## 6. Conclusions

A central challenge in the inverse design of UHPC is the generalizability of models when untested feedstocks are used. Even larger datasets may contain a limited diversity of the range of potential material components that can be encountered, and it is critical to challenge models with validation experiments outside the original dataset. Based on data aggregated from the literature on a diversity of UHPC formulations, Bayesian ML has been used to compare the accuracy of models parameterized by composition against those parameterized by latent variables related to empirical models on the factors that govern the compressive strength. Compositional GPR models had higher accuracy (*R*^2^ = 0.91) than the latent-variable models (*R*^2^ = 0.77) in training the algorithm, but significantly lower generalizability as gauged by the miscalibration areas (0.20 versus 0.06, respectively). These results indicate that while the compositional model accurately predicts the compressive strength in the regions covered by the data in the training set, the models are not as accurate across the broader response surface.

This conclusion was reinforced by the optimized compositions for UHPC mixes with compressive strengths greater than 100 MPa that incorporated a fine aggregate with a larger size than that in the training sets, where the compositional model had significantly lower accuracy (*R*^2^ = −0.06) than that of the latent-variable model (*R*^2^ = 0.67). While models parameterized by compositional variables may suffer from overconfidence and poor generalizability, these results suggest that Bayesian ML models parameterized by latent variables can be a powerful design tool for UHPC and other complex cementitious materials.

## Data Availability

The original database culled from the literature and the database including latent variables used for the machine learning work can be found via the Georgia Tech repository at: https://hdl.handle.net/ 1853/75045, https://hdl.handle.net/1853/75044.
